# Evaluating ‘living well’ with mild-to-moderate dementia: Co-production and validation of the IDEAL My Life Questionnaire

**DOI:** 10.1177/14713012231188502

**Published:** 2023-07-12

**Authors:** Linda Clare, Claire Pentecost, Rachel Collins, Anthony Martyr, Rachael Litherland, Robin G Morris, Catherine Quinn, Laura D Gamble, Serena Sabatini, Christina Victor

**Affiliations:** 3286 University of Exeter Medical School, Exeter, UK; NIHR Applied Research Collaboration South-West Peninsula, Exeter, UK; 3286 University of Exeter Medical School, Exeter, UK; 3286 University of Exeter Medical School, Exeter, UK; Innovations in Dementia CIC, Exeter, UK; Institute of Psychiatry, Psychology and Neuroscience, King’s College London, UK; Centre for Applied Dementia Studies, Bradford University, UK; Wolfson Centre for Applied Health Research, Bradford, UK; Population Health Sciences Institute 151476 , Newcastle University, UK; 3286 University of Exeter Medical School, Exeter, UK; College of Health, Medicine and Life Sciences, 3890 Brunel University London, UK

**Keywords:** Alzheimer’s, quality of life, well-being, satisfaction with life, depression, reliable change index

## Abstract

**Objectives:**

We aimed to co-produce and validate an accessible, evidence-based questionnaire measuring ‘living well’ with dementia that reflects the experience of people with mild-to-moderate dementia.

**Methods:**

Nine people with dementia formed a co-production group. An initial series of workshops generated the format of the questionnaire and a longlist of items. Preliminary testing with 53 IDEAL cohort participants yielded a shortlist of items. These were tested with 136 IDEAL cohort participants during a further round of data collection and assessed for reliability and validity. The co-production group contributed to decisions throughout and agreed the final version.

**Results:**

An initial list of 230 items was reduced to 41 for initial testing, 12 for full testing, and 10 for the final version. The 10-item version had good internal consistency and test-retest reliability, and a single factor structure. Analyses showed significant large positive correlations with scores on measures of quality of life, well-being, and satisfaction with life, and expected patterns of association including a significant large negative association with depression scores and no association with cognitive test scores.

**Conclusions:**

The co-produced My Life Questionnaire is an accessible and valid measure of ‘living well’ with dementia suitable for use in a range of contexts.

## Introduction

‘Living well’ with a chronic illness or disability means experiencing the best achievable state of physical, mental, and social health. Capability to ‘live well’ is influenced by the person’s physical, social and cultural context, and the experience of ‘living well’, unique to each individual, is reflected in ‘a self-perceived level of comfort, function and contentment with life’ (Institute of Medicine, 2012, p. 32). This concept has been applied to the experience of living with dementia and has encouraged a more positive approach to supporting people affected by the condition (Quinn et al., 2022). While the positive emphasis inherent in the concept of ‘living well’ is potentially valuable and has been useful in countering negative narratives about dementia in public discourse, it must be acknowledged that it also carries some risks (Bartlett et al., 2017). One is the risk that by taking a relentlessly positive view we fail to accept the reality of living with dementia and the suffering that people experience. Another is the risk that, if managing to ‘live well’ is implicitly seen as an individual responsibility, this reduces the likelihood that the resources needed to provide the framework of supportive services and communities on which the potential for ‘living well’ largely depends will be forthcoming. We use ‘living well’ here in the sense that people experiencing the challenges of living with dementia can and should be enabled to experience the best achievable state of health and subjective well-being, acknowledging that this requires a facilitative social and environmental context and appropriate support.

An important concept that captures key aspects of this approach to ‘living well’ is quality of life, ‘an individual’s perceptions of their position in life in the context of the culture and value systems in which they live and in relation to their goals, expectations, standards and concerns’ and affected by a person’s ‘physical health, psychological state, level of independence, social relationships, personal beliefs, and relationship… to the environment’ (WHOQOL Group, 1994, p. 28). In health research, quality of life is sometimes defined more narrowly in terms of health status or the specific effects of a health condition, but the wide-ranging impact of dementia suggests that a broad conceptualisation is more appropriate. Lawton’s model (Lawton, 1994) has been particularly influential in shaping such broader conceptualisations of quality of life in dementia and the development of quality of life measures (Ready & Ott, 2003). This model proposes that both subjective perceptions and objective elements form part of an evaluation of quality of life, with the multiple dimensions of psychological well-being, cognitive and functional ability, and features of the person’s environment and living situation all contributing to the overall picture. Other concepts capturing elements of ‘living well’ are well-being and satisfaction with life. Well-being research often distinguishes between hedonic and eudaimonic aspects (Willroth et al., 2023). Hedonic or subjective well-being involves experiencing life as enjoyable and satisfying, and is defined as a balance of positive and negative emotions and a positive appraisal of one’s current situation (Diener & Chan, 2011). Eudaimonic well-being refers to living in a way that is consistent with one’s potential, and includes components of self-acceptance, autonomy, environmental mastery, positive relations, purpose in life and personal growth (Willroth et al., 2023). Closely related to this, satisfaction with life encompasses having a sense of meaning and purpose, feeling in control, participating socially, and experiencing personal growth and happiness (St John & Montgomery, 2010). A recent scoping review argues for an asset- or strengths-based framework for understanding well-being and satisfaction with life among people with dementia, emphasising psychological, emotional and social well-being (Clarke et al., 2020).

Where promoting capability to ‘live well’ is the aspiration, it is important to evaluate whether progress is being made towards this goal. This requires a means of measuring ‘living well’ that is accessible and suitable for a variety of contexts. Although no measure specifically refers to ‘living well’, numerous measures are used in research to measure quality of life and related constructs. In a systematic review of factors associated with quality of life, well-being, and satisfaction with life in dementia, we identified 45 quality of life measures, seven well-being measures and four measures of satisfaction with life (Martyr et al., 2018). While some contain items developed through interviewing people with dementia, and the opinions of people with dementia have increasingly been taken into account in measure development (Missotten et al., 2016), most measures have been developed with little or no direct input from people with dementia (Bowling et al., 2015). Hence, these measures may not necessarily cover what is most important to respondents.

Evidence about what ‘living well’ means to people with dementia, and what is important for ‘living well’, gathered in the Improving the experience of Dementia and Enhancing Active Life (IDEAL) cohort study offers a basis for developing an accessible measure. Responses to the open-ended question ‘what does living well mean to you?’ were provided by 1,314 cohort participants with mild-to-moderate dementia (Quinn et al., 2022), and were considered alongside results of statistical modelling to create a comprehensive picture. Responses to the open-ended question showed that participants perceived ‘living well’ as involving an engaged and active lifestyle covering psychological (positive outlook on life, sense of purpose, able to cope), social (living situation and environment, security), relational (good relationships), physical (able to get out and about) domains and ability to manage everyday life (independence, getting on with life) (Quinn et al., 2022). Self-ratings by people living with dementia on questionnaire measures of quality of life, well-being and satisfaction with life were used to create a composite ‘living well’ score and examine associations with psychological, social, relational, and physical health and ability to manage everyday life. Statistical modelling confirmed that all these domains were important for ‘living well’, with the psychological domain most closely linked to the subjective experience of ‘living well’ (Clare et al., 2019; Clare et al., 2022a; Clare et al., 2022b). Discussions with the ALWAYs group, the IDEAL Patient and Public Involvement and Engagement (PPIE) group of people with dementia and carers (Litherland et al., 2018), indicated that, taken together, these findings resonated with their experience and covered the topics that were important to them.

This evidence provides a basis for developing a measure of ‘living well’ that reflects the experience of people living with dementia, but to ensure that the resulting measure is relevant, accessible, and suitable for use in a range of contexts, it is essential to involve people with dementia in the process of measure development. The development process should yield a measure that is not only relevant and accessible but also has satisfactory psychometric properties and shows expected patterns of associations; for example, it should have strong positive correlations with scores on measures of quality of life and on measures of related or contributing constructs such as well-being and satisfaction with life, and similar patterns of associations with other relevant factors. In this study we set out to involve people with dementia in co-producing a measure that meets these criteria, building on evidence from the IDEAL cohort. We aimed to:

• Co-produce with people living with dementia a questionnaire measure of ‘living well’ that reflects their experience.
• Demonstrate reliability and validity of the questionnaire through inclusion in structured interviews with IDEAL cohort participants living with mild-to-moderate dementia.

## Methods

The study was conducted in three stages: initial co-production, preliminary testing, and validation. Ethical approvals were provided by the University of Exeter Psychology Ethics Committee, reference eCLESPsy000569 and 001802, and Wales Research Ethics Committee 5, reference 18/WS/0111. All participants provided informed consent.

### Initial Co-Production

A co-production group of nine volunteers living with dementia was formed. Volunteers were identified through an invitation to members of the DEEP network of peer support groups for people with dementia. The volunteers who joined the co-production group were three men and six women, all white British, ranging in age from 61 to 87 years (mean 69.0, SD 8.44). Eight were diagnosed with Alzheimer’s disease and one with frontotemporal dementia. All were living with mild-to-moderate dementia and time since diagnosis ranged from 2 to 13 years. The group was for people with dementia only, and all members were able to participate independently in facilitated group sessions. Most travelled to and from the sessions independently, but three required door-to-door transport for which taxis were provided, and for two members the facilitator liaised with a family carer about travel arrangements. In one case the facilitator contacted the family carer after the meetings to explain what had taken place.

Group members met with two IDEAL research team members for three full-day and five half-day workshops in Manchester, UK. Six workshops were held between January and March 2019 and two in September and October 2019; a third researcher joined the last two meetings. Group members chose to name themselves the Dementia Experts into Action Research (DEAR) Group. They worked on the format and the content of the new questionnaire during the workshops and between meetings and continued to contribute throughout the initial testing and validation phases.

### Initial Testing of Questionnaire Items

Initial testing of the longlisted items was incorporated into the round of telephone or video-call data collection conducted with the IDEAL cohort between September 2020 and March 2021, during the COVID-19 pandemic (Clare et al., 2022c; Sabatini et al., 2022). To limit participant burden, items were divided into two blocks, with participants asked to complete one or other block in randomly determined order. Where the interviewer deemed it appropriate, participants completing one block were invited to also complete the second block. The interviewer introduced the questionnaire items by saying ‘I’d like to know how much you agree or disagree with the following statements’.

The target minimum sample size was 30 participants (Perneger et al., 2015). To identify a shortlist of items for further testing, analyses focused on item characteristics, frequency of missing responses, frequency of endorsement of response options, and bivariate correlations between items. Results were considered in the context of DEAR group opinions and a shortlist of items was derived.

### Validation Study

The shortlisted items were included in the sixth wave of data collection with the IDEAL cohort, conducted over two telephone or video-call sessions between April and December 2021. The target minimum sample size was 100, selected to ensure sufficient data for confirmatory factor analysis (Kline, 1979; MacCallum et al., 1999) and analyses of internal consistency (Bujang et al., 2018) and criterion validity (Trigg, Jones, & Skevington, 2007; Trigg, Skevington, & Jones, 2007). For test-retest reliability the target minimum sample size was 30 (Lobban et al., 2005; Quinn et al., 2018; Trigg, Jones, & Skevington, 2007; Trigg, Skevington, & Jones, 2007). The items were included in the first session, and to assess test-retest reliability participants were invited to complete the items again in the second session if they wished. The interviewer introduced the items by saying ‘I am going to read out a series of statements and I would like you to tell me how much you agree or disagree with each one.’ After presenting all items, the interviewer asked the participant for feedback about the items.

Information about demographic characteristics, dementia diagnosis and co-morbid conditions (Charlson et al., 2008) was taken from study records. Data from the following measures administered during the structured interview were used in the analyses:

• Quality of Life in Alzheimer’s Disease Scale (Logsdon et al., 2000) is a 13-item scale on which respondents rate aspects of their current situation such as memory, mood, health, functional ability, and overall quality of life on a 4-point Likert-type scale. Possible scores range from 13 to 52, with higher scores indicating better quality of life.
• World Health Organization-Five Well-Being Index (Bech, 2004) is a 5-item scale on which respondents rate their level of psychological well-being in relation to mood, activity and interests using a six-point Likert-type scale. Possible scores range from 0 to 25, with higher scores indicating greater well-being; scores can be transformed to a percentage scale.
• Satisfaction with Life Scale (Diener et al., 1985) is a 5-item scale on which respondents rate their satisfaction with aspects of life on a 7-point Likert-type scale. Possible scores range from 5 to 35 with higher scores reflecting greater satisfaction.
• Subjective health (Bowling, 2005) is a single question asking ‘Overall, how would you rate your health in the past four weeks?’ with responses on a 6-point Likert-type scale. Possible scores range from 1 to 6 with higher scores indicating a more positive evaluation. Better subjective health was associated with higher scores for quality of life, well-being and satisfaction with life in the IDEAL cohort (Clare et al., 2019).
• Self-continuity (Clare et al., 2020) is a single item asking respondents to indicate the extent to which they agree with the statement ‘I am still the same person as I have always been’ on a 5-point Likert-type scale. Possible scores range from 0 to 4 with higher scores indicating greater agreement. Discontinuity was associated with lower scores for quality of life, well-being and satisfaction with life in the IDEAL cohort (Clare et al., 2020).
• Geriatric Depression Scale 10-item version (Almeida & Almeida, 1999). This short-form version elicits binary responses. Possible scores range from 0 to 10 with scores of four or above indicating depression. The correlation between Geriatric Depression Scale 10 scores and quality of life was −0.436 in our systematic review (Martyr et al., 2018) and depression was associated with lower scores for quality of life, well-being and satisfaction with life in the IDEAL cohort (Clare et al., 2019).
• De Jong-Gierveld Loneliness Scale (De Jong Gierveld & Tilburg, 2006). This six-item scale contains three items covering emotional loneliness (maximum score 3) and three items covering social aspects of loneliness (maximum score 3). Total scores range from 0–6; scores of 0–1 are classed as not lonely, 2–4 as moderately lonely, and 5–6 as severely lonely. Loneliness was associated with lower scores for quality of life, well-being, and satisfaction with life in the IDEAL cohort (Clare et al., 2019; Victor et al., 2020).
• Montreal Cognitive Assessment 5-minute version (Wong et al., 2009). The score range is 0 to 30, with lower scores indicating greater cognitive impairment. Scores can be converted to a Mini-Mental State Examination (Folstein et al., 1975) equivalent (Wong et al., 2018). Cognition was not associated with quality of life in our systematic review (*r* = 0.081) (Martyr et al., 2018) or in the IDEAL cohort (Clare et al., 2019).

Analyses first involved scrutiny of responses to, and participant feedback on, individual items. Reliability was assessed in terms of internal consistency and test-retest reliability. Measure structure was explored through confirmatory factor analysis. Content validity was examined by relating item content to the domains of the IDEAL ‘living well’ model and the themes represented in dementia-specific measures of quality of life (Missotten et al., 2016). Criterion validity was assessed in relation to self-ratings on other relevant measures and cognitive test performance from the same wave of data collection. We considered correlation coefficients ≤0.09 as negligible, 0.10–0.29 as small, 0.30–0.49 as moderate, and ≥0.50 as large (Cohen, 1988). Final decisions on presentation, layout and title were taken by the DEAR group.

## Results

### Co-Production

The DEAR group members reviewed and completed examples of questionnaires assessing similar constructs. Facilitators provided practical support for completing questionnaires by reading out the questions for members who had difficulty with reading, and by writing down the responses for members who had difficulty with writing. The group members discussed their reactions to the questionnaires and identified key parameters for the new questionnaire, including accessibility, brevity and a limited set of response options, and a focus on feelings at the time of completion rather than over a defined prior period. They opted for a five-point response scale with a neutral option (strongly agree, agree, neither agree nor disagree, disagree, strongly disagree), decided to exclude negatively worded items, and chose not to include specific reference to dementia or symptoms. Group members felt that the questionnaire should focus on how people perceive their life while living with dementia, rather than on the experience of dementia itself.

The group explored IDEAL findings about ‘living well’ with dementia and looked in detail at responses to the open-ended question ‘what does living well mean to you?’ (Quinn et al., 2022). By initially discussing their own experiences of living with dementia, the group built on their personal perceptions to create shared knowledge and different understandings. The group felt that the domains and categories identified in the IDEAL research covered the full range of relevant topics and reflected their personal experience of living with dementia. They began to generate suggestions for possible questionnaire items. A working group of eight research team members also generated possible items based on the responses to the open-ended question, keeping as closely as possible to the wording used by respondents. An initial list of 230 items was reduced through three iterative rounds of reviewing to 122. For each of these 122 items DEAR group members indicated whether it should be kept or discarded, and where relevant suggested possible amendments. On this basis, 41 items were selected for initial testing and divided into two blocks.

### Initial Testing

Fifty-three people with dementia completed at least one item block. 30 completed both blocks, 12 completed block one (items 1 to 20) only and 11 completed block two (items 21 to 41) only. Consequently, there were 42 responses for items 1 to 20 and 41 responses for items 21 to 41. There were few instances of missing data; three items each had one instance of missing data, all from different participants.

For each of the 41 items we considered statistical information about frequency of endorsement and correlations with other items alongside the opinions of DEAR group members about the importance and suitability of the item. DEAR group members strongly recommended removing four items asking about negative aspects of experience such as fear or worry because they considered it preferable to elicit difficulties in the form of disagreement with positive statements. For the remaining 37 items, we examined bivariate correlations between items and explored frequency of endorsement across response options to identify items where the sum of responses to two adjacent options was ≤10% of the total number of responses. Six items had Spearman’s rho correlation coefficients >0.75 with at least one other item, and in four cases there was overlap with two other items; in all six cases most participants answered agree or strongly agree. All six of these items were removed. Among the remaining 31 items, there were 22 items where most participants answered agree or strongly agree, but no items showed high endorsement of the neutral option. Balancing DEAR group opinions and data on frequency of endorsement led to identification of a set of 12 items for further testing. Feedback was sought from the DEAR group and ALWAYs group. With one minor change agreement was reached on the 12-item set.

### Validation Study

There were 136 people with dementia who responded to the 12 items, and 64 of these participants gave additional feedback. To assess test-retest reliability, 45 participants agreed to respond to the items again in the second session, conducted approximately 1 week later (median 7 days; mean 6.56 (1.80) days; range 4 to 14 days).

Participant characteristics for the full sample and retest sample are summarised in Table 1, and scores on study assessments and self-reported measures for the full sample in Table 2. The 5‐minute Montreal Cognitive Assessment scores ranged from 1.5 to 30, approximately equivalent to a Mini-Mental State Examination score range of 10 to 30, indicating that participants were in the mild-to-moderate stages of dementia. See Supplementary Table 1 for details of retest group scores on study assessments. There were hardly any missing data; one participant gave no response to a single item, precluding calculation of a total score. Frequency of endorsement showed that the median response to all items was ‘agree’, but all five response options were endorsed for nine of the 12 items. Kurtosis was considered satisfactory (<3) for all but one item, which also had the lowest item-total correlation (*r* = 0.351). Other item-total correlations ranged from *r* = 0.424 to *r* = 0.714. Test-retest correlations for individual items ranged from *r* = 0.378 to *r* = 0.809.

**Table 1.** Characteristics of Validation Study Participants.

	Whole sample (n = 136)	Retest sample (n = 45)
Age mean (SD)	74.52 (9.93); range 51–99	74.47 (10.47); range 51–92
Sex n (%): Men	79 (58.1)	21 (46.7)
Women	56 (41.2)	24 (53.3)
Ethnicity n (%): White British	126 (92.6)	44 (97.8)
White other	3 (2.2)	—
Other	2 (1.5)	1 (2.2)
Missing	5 (3.7)	—
Marital status n (%): Single	4 (2.9)	2 (4.4)
Married/partner/cohabiting	96 (70.6)	29 (94.4)
Divorced/separated	20 (14.7)	8 (17.8)
Widowed	16 (11.8)	6 (13.3)
Living situation n (%): Live alone	32 (23.5)	13 (28.9)
Live with spouse/partner	98 (72.1)	29 (64.4)
Live with other	6 (4.4)	3 (6.7)
Dementia type n (%): Alzheimer’s	63 (46.3)	24 (53.3)
Vascular dementia	15 (11.0)	4 (8.9)
Mixed	20 (14.7)	4 (8.9)
Frontotemporal dementia	20 (14.7)	7 (15.6)
Parkinson’s disease dementia	5 (3.7)	—
Dementia with Lewy bodies	8 (5.9)	6 (13.3)
Unspecified/other	5 (3.7)	—
Education n (%): No qualifications	26 (19.1)	9 (20.0)
School leaving age 16	27 (19.9)	12 (26.7)
School leaving age 18	53 (39.0)	17 (37.8)
University	29 (21.3)	7 (15.6)
Missing	1 (0.7)	—
Co-morbid conditions mean (SD)	2.32 (1.97); range 0–9; n = 135	1.91 (1.78); range 0–7; n = 44

**Table 2.** Participants’ Scores on Study Assessments and Self-Rated Measures.

Measure	Score range	Mean	SD	Range	n
5-minute Montreal Cognitive Assessment	0–30	20.01	7.02	1.50–30	136
Estimated Mini-Mental State Examination	0–30	25.15	5.17	10–30	136
Neuropsychiatric Inventory Questionnaire – number of symptoms, informant rated	0–12	3.90	2.16	0–9	102
Quality of Life in Alzheimer’s Disease	13–52	35.12	5.96	19–48	136
Satisfaction with Life Scale	5–35	23.82	7.30	5–35	136
World Health Organization-Five Well-Being Index	0–100	51.12	23.82	0–96	136
Subjective health single question	1–6	3.40	1.20	1–6	136
Self-continuity single question	1–5	3.50	1.29	1–5	135
Geriatric Depression Scale 10-item version	0–10	3.10	2.24	0–10	136
De Jong-Gierveld Loneliness Scale	0–6	2.18	1.70	0–6	136

Of the 64 people who commented, 31 said the questions were relevant, clear, and easy to understand. Only two said they found the questions difficult to answer overall, although seven people found one question hard to understand. Some noted that their answers would change depending on how they felt on a given day and in relation to current circumstances.

**Figure 1.**
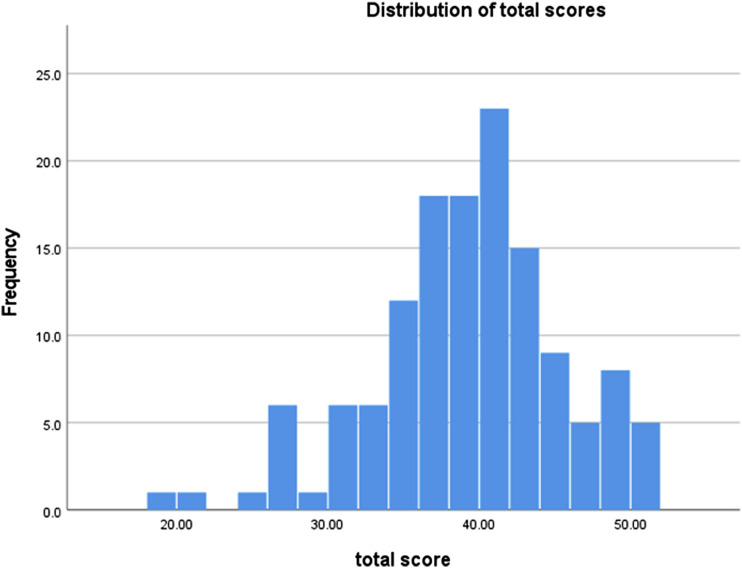
Distribution of total scores on the My Life Questionnaire in the validation study (n = 135). Possible scores range from 10 to 50.

The results for the 12-item set therefore identified one item with high kurtosis and the lowest item-total correlation, and one item that appeared difficult for some participants to understand. These two items were removed, leaving 10 items. For the 10-item version of the questionnaire, possible scores ranged from 10 to 50 and the mean score was 38.76 (s.d. 6.18; range 19–50; median 39; mode 40). Figure 1 shows the distribution of total scores. Internal consistency was good (Cronbach’s alpha 0.815), and test-retest reliability was satisfactory (*r* = 0.755, *p* < 0.001). The frequency of endorsement of each response option for each item is shown in Supplementary Figure 1. There were no statistically significant differences in mean scores according to age, sex, educational attainment or type of dementia (see Table 3).

**Table 3.** Mean Score on the My Life Questionnaire (possible score range 10–50) for the Whole Sample (n = 135) and for Sub-Groups According to Age, Sex, Educational Level and Dementia Diagnosis.

Measures	N	Mean score	SD	Range	Statistic	*p* value
Whole sample	135	38.76	6.18	19–50		
Sex						
Female	56	38.73	6.56	19–50	*t*(1,134)0.48	0.961
Male	79	38.79	5.94	21–50		
Age group						
<65	21	38.76	7.77	19–50	*F*(5,129)0.98	0.433
65–69	21	40.38	5.01	32–50		
70–74	25	39.68	6.41	21–50		
75–79	25	37.04	5.17	26–49		
80–84	19	39.42	5.56	27–50		
85+	24	37.67	6.72	26–49		
Educational level						
No qualifications	26	39.42	6.45	21–50	*F*(3,131)0.32	0.809
School leaving age 16	27	38.56	7.50	24–50		
School leaving age 18	53	38.23	5.82	19–50		
University	29	39.35	5.42	26–50		
Dementia diagnosis						
Alzheimer’s disease	63	39.13	6.20	21–50	*F*(6,128)1.63	0.145
Vascular dementia	15	39.73	7.29	26–48		
Mixed Alzheimer’s and vascular	20	36.50	4.92	27–48		
Frontotemporal dementia	20	41.30	5.78	31–50		
Parkinson’s disease dementia	5	36.20	2.59	33–40		
Dementia with Lewy bodies	8	36.38	8.12	19–44		
Unspecified/other	4	36.00	3.83	31–39		

Measure structure was examined with confirmatory factor analysis after confirming suitability of the data (Kaiser-Meyer-Olkin measure of sampling adequacy 0.861; Bartlett’s Test of Sphericity chi-square = 376.68, df = 45, *p* < 0.001). Due to the ordinal nature of the items, a polychoric correlation matrix was used to fit the factor analysis using maximum likelihood estimation. Parallel analysis and the scree plot indicated that a single factor solution was optimal and explained 45% of the variance. Factor loadings are shown in Supplementary Table 2.

Criterion validity was examined through exploration of associations with self-ratings on other relevant measures. There was a significant large positive association with Quality of Life in Alzheimer’s Disease scores (*r* = 0.683, *p* < 0.001, n = 135). There were significant large positive associations with World Health Organization-Five Well-Being Index scores (*r* = 0.538, *p* < 0.001, n = 135), Satisfaction with Life Scale scores (*r* = 0.543, *p* < 0.001, n = 135), and subjective health (*r* = 0.553, *p* < 0.001, n = 135), and a significant moderate positive association with self-continuity (*r* = 0.405, *p* < 0.001, n = 134). There was a significant large negative association with Geriatric Depression Scale 10 score (*r* = 0.578, *p* < 0.001, n = 135) and a significant moderate negative association with De Jong-Gierveld Loneliness Scale score (*r* = 0.416, *p* < 0.001, n = 135). Total score was not significantly correlated with Montreal Cognitive Assessment 5‐minute version scores (*r* = −0.102, *p* = 0.240, n = 135).

Content validity was checked by relating the items to the domains in the IDEAL ‘living well’ model and the themes covered in dementia-specific quality of life measures (Missotten et al., 2016). Although some items were difficult to classify into a single domain and might have been grouped under more than one heading, all domains and themes appeared to be covered.

A final round of consultation was held with the DEAR group, now meeting online due to the COVID-19 pandemic. Three of the original members decided not to continue at this stage. One had said after the first round of workshops that although he had enjoyed the meetings he felt he had done enough and did not want to continue, and two did not want to move to meeting online. The remaining six original members were joined by three new members, two men and one woman, all in their 60s and living with mild or moderate Alzheimer’s disease or frontotemporal dementia. Family carers often provided background assistance with technology to support online participation, but they did not take part in the meetings.

The group made decisions about the title, format and layout of the questionnaire, which was named the My Life Questionnaire. The questionnaire is free to use and a printable copy is included in the Living with Dementia Toolkit https://livingwithdementiatoolkit.org.uk/home/how-are-you-feeling-today/ and can also be downloaded here: https://medicine.exeter.ac.uk/reach/publications/. A reproduction of the printable version is included in the supplementary material; see Supplementary Figure 2.

## Discussion

This paper reports the co-production and validation of the IDEAL programme My Life Questionnaire for people with mild-to-moderate dementia, an accessible measure capturing the extent to which people with dementia feel they are ‘living well’ and experiencing a good quality of life. This is the first questionnaire of its kind to be co-produced and validated by people with dementia working together with researchers. It is grounded in understanding of what people with dementia themselves consider important for ‘living well’ and in evidence from the IDEAL cohort. It combines face and content validity with sound psychometric properties, evidenced through good internal reliability and criterion validity and demonstration of a single factor structure. Scores are strongly correlated with scores on research-based measures of quality of life, well-being, and satisfaction with life. My Life is suitable for self-completion and could be used in a range of contexts including community settings and surveys, providing high-quality, reliable data about capability to ‘live well’ with dementia, and complementing existing research-based quality of life measures.

The co-production process was central to measure development and was particularly important in view of the intended purpose of the questionnaire. The role of people with dementia as PPIE representatives in research is well-established, and there is increasing acknowledgement of the value of involving people with dementia in co-designing products and services (Wang et al., 2019) such as assistive technology or telehealth applications (Hanson et al., 2007; Meiland et al., 2012; Robinson et al., 2009) or events to promote public understanding (Phillipson et al., 2019). However, we found no previous examples of co-producing a standardised questionnaire.

The central tenet of co-production emphasises people with dementia and researchers sharing power and responsibility for the project and the knowledge generated (Hickey, 2018). Defining and understanding the nature and implications of the role was an initial challenge for group members and a frequent topic of discussion in the early meetings, as people moved from broad discussions of what ‘living well’ with dementia means to acting as co-researchers working towards developing a tangible product. As the work progressed, the role became more familiar, and members became adept at reminding each other about what the group was aiming to achieve. For their part, research team members benefitted from being challenged to question assumptions and think flexibly about construction and format of the measure. For example, co-production discussions questioned the assumption that negatively worded items should be included to provide balance. Reviewing relevant literature indicated that including both positively and negatively worded items increases level of difficulty (Melnick & Gable, 1990), reduces reliability (Barnette, 2000) and tends to yield a two-factor scale (Horan et al., 2003; Lindwall et al., 2012), supporting the preference of the co-production group for including only positively-phrased items.

The timescale involved in progressing from initial discussions to the final validated measure was a particular concern when involving co-production group members living with a progressive condition. This was something we considered and discussed with the group from the outset. Because of the nature of the project, periods where the focus was on co-production activities were interspersed with periods during which the research team were collecting data, with the timing of data collection determined by the data collection schedule of the wider cohort study. It was important to prepare the group for what to expect and to keep them in touch and updated throughout the data collection stages, especially as in the event data collection was delayed by over a year due to capacity issues in the main study and the impact of the COVID-19 pandemic, which in turn meant that group members had to adjust to meeting online. The facilitator stayed in touch with everyone and provided updates about the delays but noticed that some members’ abilities had declined markedly during the pandemic, so that it was hard for them to remember their role in the group and the work the group had been doing. Face-to-face meetings had provided a rich context for the project and a regular routine, with the same meeting room, the same time of day and the same lunch, which meeting online could not replace. It was encouraging that despite these challenges two-thirds of the original members continued to contribute throughout the project, and that some then chose to extend their involvement with the IDEAL research programme by joining the ALWAYs group.

The content of the measure was based on the views and experiences of people with dementia, and face validity is supported by the effective coverage of themes identified in a review of dementia-specific quality of life measures (Missotten et al., 2016): affect, activity, interactions, self-concept or self-esteem, cognition, physical health, psychological and behavioural aspects, independence, attachment and living conditions. Similarly, there is evident synergy with the domains of well-being proposed in the review by Clarke et al. (2020): psychological well-being, which includes positive self-concept and a sense of meaning and purpose; emotional well-being, reflecting positive emotional states; and social well-being, reflecting connections and belonging. The content is also consistent with qualitative studies exploring what people with dementia feel is important for their quality of life, which refer to relationships and communication, activity and independence, environment and home, health and sleep (Harding et al., 2019; Trigg, Skevington, & Jones, 2007; Williamson, 2010), mood (Trigg, Skevington, & Jones, 2007) and identity (Williamson, 2010). Although developed by and for people with dementia, the measure contains nothing that is specific to the experience of living with dementia; it does not for example ask about memory difficulties or other symptoms. It could, therefore, be tested to determine whether it is also suitable for people living with some other types of disability or for older people more generally.

The study has some limitations. While sample sizes were sufficient for most analyses, in the initial testing phase the number of responses available per item was relatively small for the frequency of endorsement analysis (Streiner et al., 2015). Further work could re-examine data from that phase or collect additional data for the 41-item set and apply Rasch analysis to refine the measure, acknowledging that items requiring responses about the extent of agreement or disagreement are not all equally ‘agreeable’ (Boone, 2016). The sample for the validation study included men and women with a good distribution of ages, educational backgrounds, living situations and other health conditions, whose responses on the self-rated measures used in the validation analyses covered the full range of possible options. The distribution of dementia diagnoses in the sample was slightly different to population estimates (Prince et al., 2014) due to over-sampling of people with rarer dementias, but Alzheimer’s, vascular and mixed dementia still accounted for nearly three-quarters of all diagnoses. While the proportion of individuals from minority ethnic groups in the IDEAL cohort was consistent with British population estimates (Pham et al., 2018; Prince et al., 2014) absolute numbers were small, and further work could be undertaken to consider possible cultural differences in responses. For the sub-group analyses we were able to undertake, limited numbers in some categories leave open the possibility that significant differences might have been missed. For example, non-significant differences in mean score according to type of dementia were consistent with reliable differences seen in Quality of Life in Alzheimer’s Disease data from the whole IDEAL cohort (Wu et al., 2018), including markedly lower scores among people with Parkinsonian dementias. Nevertheless, the inclusion of people with different types of dementia demonstrates that the measure is useful across diagnostic categories as well as across groups defined by age, sex and educational level. People with a Mini-Mental State Examination equivalent score of 10, the lowest score in the validation sample, were able to complete the measure.

Users of the measure may want to answer various kinds of questions. To understand the extent to which groups or individuals are ‘living well’, the validation data from the IDEAL cohort presented here provides a point of comparison, and accumulation of further data will provide a more comprehensive picture. Another potential focus of interest would be to identify which groups or individuals have poor capability to ‘live well’ in order to provide targeted support. Typically, quality of life measures do not specify cut-offs to differentiate poor from satisfactory quality of life. However, about two-thirds (68%) of scores will fall within one standard deviation of the mean, which in the validation sample approximately covered the score range 32 to 44. Scores either side of this range could be considered low or high. There could be a slight ceiling effect at the upper end of the scale, but this was considered unproblematic given that concern would focus on those with poor rather than excellent capability to ‘live well’. At the individual level, a review of responses to each item could serve as a basis for identifying challenges and exploring support needs. At the group level, to assess whether change in scores over time, for example after the introduction of a new service or intervention, is meaningful or simply the result of measurement error, a reliable change index can be calculated for the given sample (Evans et al., 1998).

## Conclusions

My Life is to the best of our knowledge the first example of a co-produced standardised questionnaire developed through collaboration between people living with dementia and researchers. This collaboration has yielded an evidence-based, validated measure of ‘living well’ for people with mild-to-moderate dementia that captures aspects of quality of life, well-being and satisfaction with life, and is accessible and suitable for use in a range of contexts, especially those where standard research instruments may be less appropriate. Further work will continue to elucidate the properties and uses of the My Life Questionnaire.

## Supplemental Material

Supplemental Material - Evaluating ‘living well’ with mild-to-moderate dementia: Co-production and validation of the IDEAL My Life Questionnaire Supplemental Material for Evaluating ‘living well’ with mild-to-moderate dementia: Co-production and validation of the IDEAL My Life Questionnaire by Linda Clare, Claire Pentecost, Rachel Collins, Anthony Martyr, Rachael Litherland, Robin G Morris, Catherine Quinn, Laura D Gamble, Serena Sabatini, and Christina Victor in Dementia: the international journal of social research and practice

## Data Availability

The My Life Questionnaire is free to use. A printable version can be downloaded from the Living with Dementia Toolkit https://livingwithdementiatoolkit.org.uk/home/how-are-you-feeling-today/ or via this link https://medicine.exeter.ac.uk/reach/publications/. Requests for further information about the IDEAL My Life Questionnaire may be directed to IDEAL@exeter.ac.uk. IDEAL cohort data were deposited with the UK Data Archive in April 2020. Details of how to access the data can be found here: https://reshare.ukdataservice.ac.uk/854293/.
