# Supplementary material for: Evaluating ‘living well’ with mild-to-moderate dementia: Co-production and validation of the IDEAL My Life Questionnaire
Source: Dementia (London). 2023 Jul 12;22(7):1548–66. doi: 10.1177/14713012231188502 (PMC10966933; doi:10.1177/14713012231188502)
Supplement: Supplemental Material - Evaluating ‘living well’ with mild-to-moderate dementia: Co-production and validation of the IDEAL My Life Questionnaire [file sj-pdf-1-dem-10.1177_14713012231188502.pdf]

# Evaluating ‘living well’ with mild-to-moderate dementia: co-production and validation of the IDEAL My Life Questionnaire

## Supplementary data

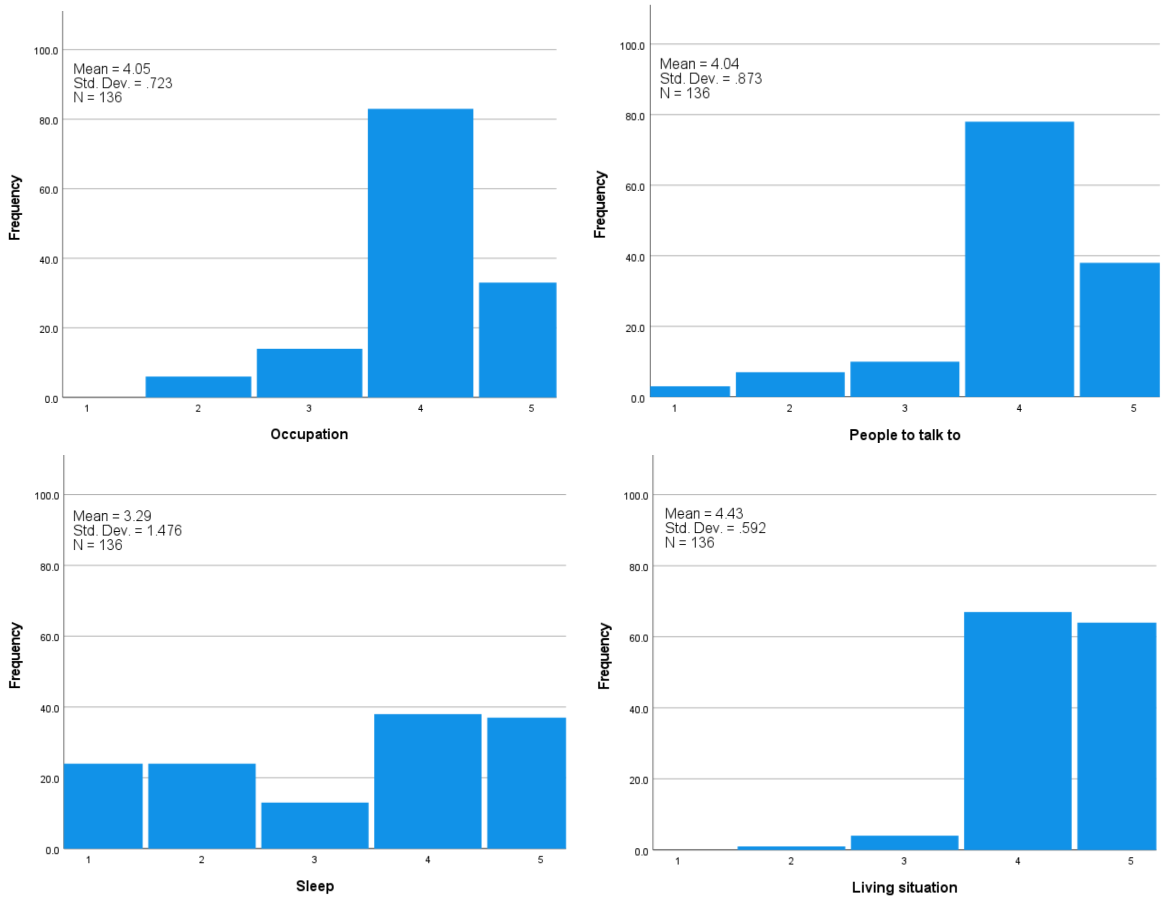

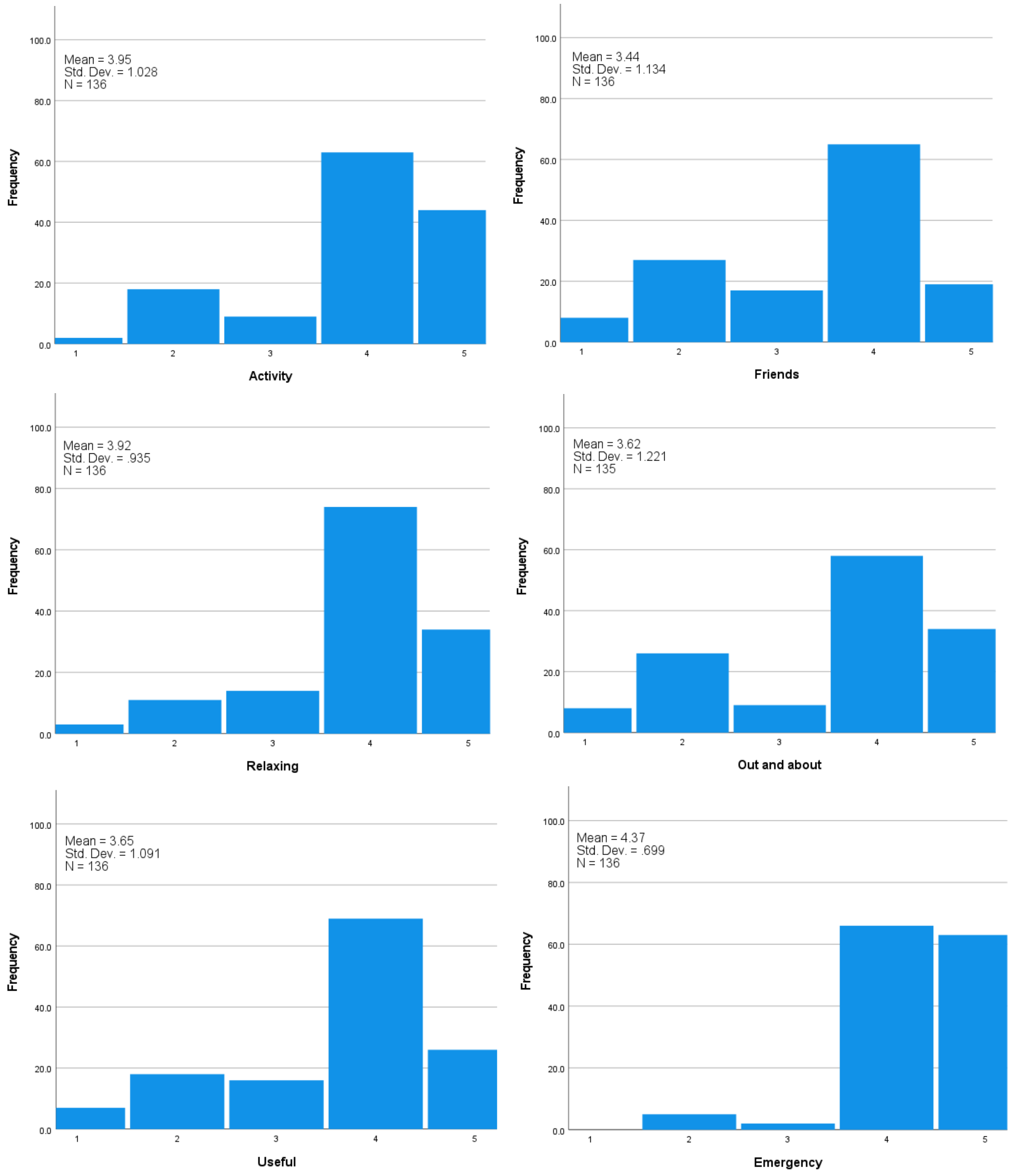


Supplementary Figure 1. Histograms showing the frequency of endorsement of each response option for each of the 10 items that make up the My Life Questionnaire. 1 = strongly disagree, 5 = strongly agree.

Supplementary Table 1. Retest group scores on study assessments

| Measure | Score range | Mean | SD | Range | n |
|---|---|---|---|---|---|
| 5-min Montreal Cognitive Assessment | 0-30 | 21.50 | 5.83 | 7.50-30 | 45 |
| Estimated Mini-Mental State Examination | 0-30 | 26.29 | 4.10 | 15-30 | 45 |
| Neuropsychiatric Inventory Questionnaire – number of symptoms, informant rated | 0-12 | 3.61 | 2.41 | 0-9 | 28 |

Supplementary Table 2. Confirmatory factor analysis of the 10 item My Life Questionnaire

| Item | Factor loading |
|---|---|
| Occupation | .649 |
| People to talk to | .761 |
| Sleep | .390 |
| Living situation | .596 |
| Activity | .741 |
| Friends | .687 |
| Relaxing | .603 |
| Out and about | .745 |
| Useful | .704 |
| Emergency | .722 |

Model fit statistics: Chi-square (df) 58.97 (35) $p = 0.007$, CFI 0.931, TLI = 0.911, RMSEA = 0.071 (df: degrees of freedom, CFI: Comparative Fit Index, TFI: Tucker-Lewis Index, RMSEA: Root Mean Square Error of Approximation).

Supplementary Figure 2. My Life Questionnaire

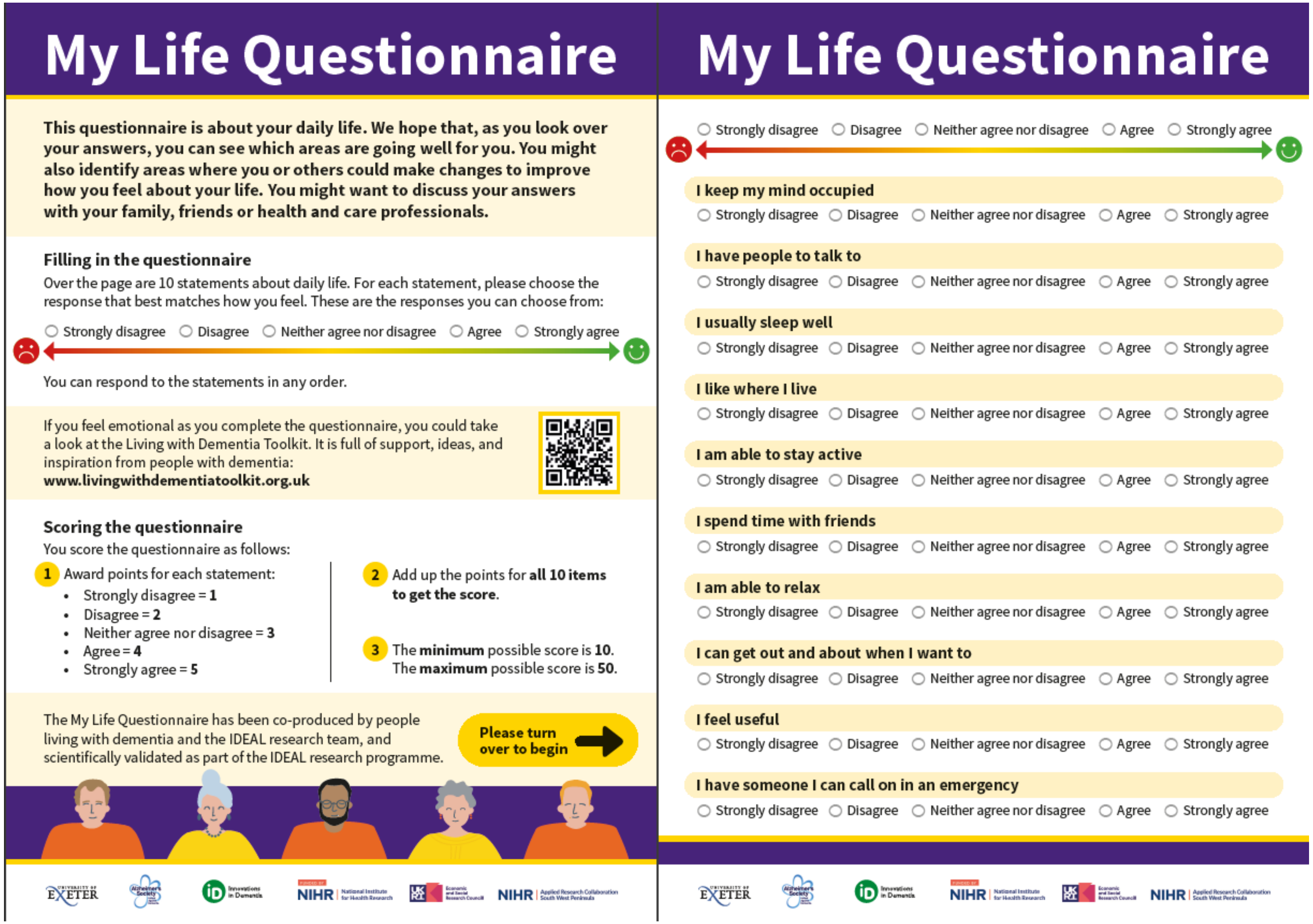

# My Life Questionnaire

**This questionnaire is about your daily life. We hope that, as you look over your answers, you can see which areas are going well for you. You might also identify areas where you or others could make changes to improve how you feel about your life. You might want to discuss your answers with your family, friends or health and care professionals.**

## Filling in the questionnaire

Over the page are 10 statements about daily life. For each statement, please choose the response that best matches how you feel. These are the responses you can choose from:

○ Strongly disagree ○ Disagree ○ Neither agree nor disagree ○ Agree ○ Strongly agree

You can respond to the statements in any order.

If you feel emotional as you complete the questionnaire, you could take a look at the Living with Dementia Toolkit. It is full of support, ideas, and inspiration from people with dementia: **www.livingwithdementiatoolkit.org.uk**

## Scoring the questionnaire

You score the questionnaire as follows:

1. Award points for each statement:
   - Strongly disagree = **1**
   - Disagree = **2**
   - Neither agree nor disagree = **3**
   - Agree = **4**
   - Strongly agree = **5**
2. Add up the points for **all 10 items to get the score.**
3. The **minimum** possible score is **10**. The **maximum** possible score is **50**.

The My Life Questionnaire has been co-produced by people living with dementia and the IDEAL research team, and scientifically validated as part of the IDEAL research programme.

**Please turn over to begin**

# My Life Questionnaire

○ Strongly disagree ○ Disagree ○ Neither agree nor disagree ○ Agree ○ Strongly agree

**I keep my mind occupied**
○ Strongly disagree ○ Disagree ○ Neither agree nor disagree ○ Agree ○ Strongly agree

**I have people to talk to**
○ Strongly disagree ○ Disagree ○ Neither agree nor disagree ○ Agree ○ Strongly agree

**I usually sleep well**
○ Strongly disagree ○ Disagree ○ Neither agree nor disagree ○ Agree ○ Strongly agree

**I like where I live**
○ Strongly disagree ○ Disagree ○ Neither agree nor disagree ○ Agree ○ Strongly agree

**I am able to stay active**
○ Strongly disagree ○ Disagree ○ Neither agree nor disagree ○ Agree ○ Strongly agree

**I spend time with friends**
○ Strongly disagree ○ Disagree ○ Neither agree nor disagree ○ Agree ○ Strongly agree

**I am able to relax**
○ Strongly disagree ○ Disagree ○ Neither agree nor disagree ○ Agree ○ Strongly agree

**I can get out and about when I want to**
○ Strongly disagree ○ Disagree ○ Neither agree nor disagree ○ Agree ○ Strongly agree

**I feel useful**
○ Strongly disagree ○ Disagree ○ Neither agree nor disagree ○ Agree ○ Strongly agree

**I have someone I can call on in an emergency**
○ Strongly disagree ○ Disagree ○ Neither agree nor disagree ○ Agree ○ Strongly agree

A printable version can be downloaded from the Living with Dementia Toolkit https://livingwithdementiatoolkit.org.uk/home/how-are-you-feeling-today/ or via this link https://medicine.exeter.ac.uk/reach/publications/.
